# Microfluidics Integrated Biosensors: A Leading Technology towards Lab-on-a-Chip and Sensing Applications

**DOI:** 10.3390/s151229783

**Published:** 2015-12-01

**Authors:** George Luka, Ali Ahmadi, Homayoun Najjaran, Evangelyn Alocilja, Maria DeRosa, Kirsten Wolthers, Ahmed Malki, Hassan Aziz, Asmaa Althani, Mina Hoorfar

**Affiliations:** 1 School of Engineering, University of British Columbia, Kelowna, BC V1V 1V7, Canada; george.luka@ubc.ca (G.L.); ali.ahmadi@ubc.ca (A.A.); homayoun.najjaran@ubc.ca (H.N.); kirsten.wolthers@ubc.ca (K.W.); 2 Nano-Biosensors Laboratory, Department of Biosystems and Agricultural Engineering, Michigan State University, 524 S. Shaw Lane, Room 115, East Lansing, MI 48224, USA; alocilja@msu.edu; 3 Carleton University, Department of Chemistry and Institute of Biochemistry, 225 Steacie Building 1125 Colonel By Drive Ottawa, ON K1S 5B6, Canada; maria_derosa@carleton.ca; 4 Department of Health Sciences, College of Arts and Sciences, Qatar University, 2713 Doha, Qatar; ahmed.malki@qu.edu.qa (A.M.); hassan.aziz@qu.edu.qa (H.A.); 5 Biomedical Research Center, Qatar University, 2713 Doha, Qatar; aaja@qu.edu.qa

**Keywords:** microfluidic, biosensor, lab-on-a-chip, microfluidic-based biosensor, micro total analysis systems (μTAS)

## Abstract

A biosensor can be defined as a compact analytical device or unit incorporating a biological or biologically derived sensitive recognition element immobilized on a physicochemical transducer to measure one or more analytes. Microfluidic systems, on the other hand, provide throughput processing, enhance transport for controlling the flow conditions, increase the mixing rate of different reagents, reduce sample and reagents volume (down to nanoliter), increase sensitivity of detection, and utilize the same platform for both sample preparation and detection. In view of these advantages, the integration of microfluidic and biosensor technologies provides the ability to merge chemical and biological components into a single platform and offers new opportunities for future biosensing applications including portability, disposability, real-time detection, unprecedented accuracies, and simultaneous analysis of different analytes in a single device. This review aims at representing advances and achievements in the field of microfluidic-based biosensing. The review also presents examples extracted from the literature to demonstrate the advantages of merging microfluidic and biosensing technologies and illustrate the versatility that such integration promises in the future biosensing for emerging areas of biological engineering, biomedical studies, point-of-care diagnostics, environmental monitoring, and precision agriculture.

## 1. Introduction

Biosensors are considered to be powerful analytical tools and are potentially useful for a wide range of applications ranging from drug discovery, to medical diagnostics, to food safety, to agricultural and environmental monitoring, and to security and defense [1]. A biosensor can be defined as an analytical device [1,2] that combines a biological sensitive recognition element [3] (such as antibodies, nucleic acids, enzymes, or aptamers) immobilized on a physicochemical transducer, and connected to a detector to identify the presence of one or more specific analytes [4], their concentrations, and kinetics in a sample. The specificity and selectivity of the biosensor is determined by the catalytic or affinity properties of the biological recognition element. The signal originating from the interaction between the analyte of interest and the biological recognition element is then transformed by a transducer to an optical or electrical readout [5,6]. Biosensors are more favorable, reliable, accurate, cost effective, and easy to use compared to other conventional lab-based detection techniques [7] due to their portability, reusability, real-time response, and high specificity and selectivity.

Microfluidics is considered to be a multidisciplinary technology that links several different sciences including chemistry, biochemistry, engineering, physics, micro-technology, nano-technology and biotechnology [8]. The large surface-to-volume ratio enables portability of microfluidic devices which is important for on-site testing. There are three classes of microfluidics: (i) continuous-flow; (ii) droplet-based; and (iii) digital microfluidics. Continuous microfluidic devices consist of permanently etched microchannels and peripheral devices (such as micropumps and microvalves) used to manipulate a stream of fluid in these devices [9]. Droplet-based microfluidic systems are based on creating droplets in micro-channels using two (or more) immiscible fluids (mostly) at a T-junction. Digital microfluidic systems, however, are fundamentally different as they provide motion and control of discrete droplets on an array of planar electrostatically-actuated electrodes.

In this paper, examples from the literature are presented to demonstrate the advantages of merging microfluidic and biosensor technology and illustrate the versatility that such a merging promises in the future biosensing for numerous areas of biological engineering, environmental monitoring, biomedical applications, agricultural monitoring, industrial monitoring, and point-of-care diagnostics. We have divided this review into three sections: (i) different types of biosensors (categorized based on the biological recognition elements and transducers); (ii) different types of microfluidic platforms with their advantages and disadvantages; and (iii) examples of integrated biosensors in different microfluidic platforms.

## 2. Biosensors

**Figure 1:**
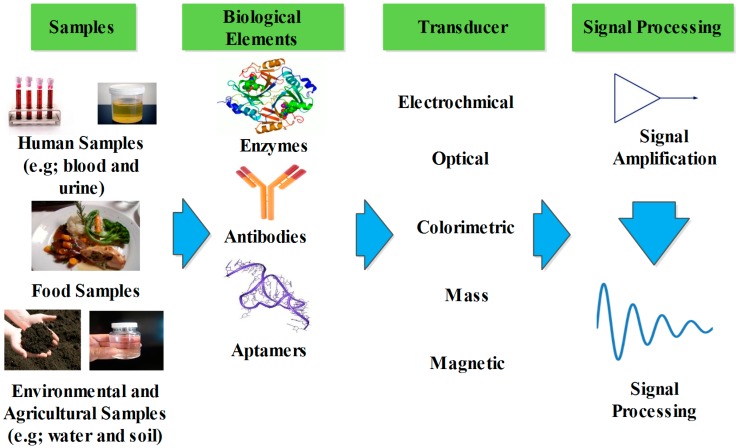
Schematic of different parts of a biosensor including biological recognition elements, transducers, and detectors.

In the past two decades, there has been a significant growth and interest in biosensor technology and research [10]. According to the International Union of Pure and Applied Chemistry (IUPAC) [11], a biosensor is defined as “*an independently integrated receptor transducer device, which is capable of providing selective quantitative or semi-quantitative analytical information using a biological recognition element*”. This makes biosensing technology a powerful analytical tool capable of detecting biological or chemical molecules using electrical [12], optical [13], or mass change readout protocols [14]. Figure 1 shows a schematic of the different parts in a biosensor. The two important parts that distinguish the biosensors are the type of biological recognition sensing element and the transducers.

### 2.1. Biosensors Categorized Based on the Type of Biological Recognition Element and Immobilization Techniques

The biological recognition sensing element dictates the selectivity and specificity that allows the biosensor to respond to a specific target or group of analytes, decreasing the possibility of interference with undesired substances [15]. The selection of the biological recognition element depends on the target of interest (e.g., antibodies and aptamers are more suitable for the detection of bacteria or pathogens; whereas enzymes are more suitable for catalytic reactions).

**Figure 2:**
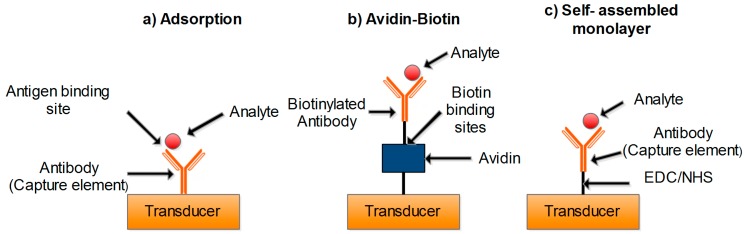
Schematic of the most common and main immobilization methods.

In general, the biological recognition elements are immobilized using different methods such as adsorption, covalent binding, entrapment, and membrane confinement. Figure 2 shows the schematic of some of the most common immobilization methods. In general, immobilization with covalent bonding is the most common and preferable method due to stability and irreversibility which prevents leakage of the biological elements from the support surface [16,17].

#### 2.1.1. Enzyme-Based Biosensors

Enzymes are proteins that have the ability to catalyze chemical reactions with a rate enhancement ranging from 10^5^ to 10^17^ greater than that of the uncatalyzed reactions [18]. Enzymes that act as biosensors typically catalyze oxidation-reduction (or redox) reactions. The turnover of these enzymes can be monitored by a variety of electrochemical methods, which makes them ideal biosensors. For example, glucose oxidase, one of the most widely-used biosensors, is an oxidoreductase enzyme that transfers electrons from glucose to molecular oxygen. The glucose biosensor, first described by Clark and Lyons in 1962 [19], uses immobilized glucose oxidase to determine glucose concentrations in bodily fluids.

One of the advantages of using enzymes as a biological recognition element in biosensor technology is that they are highly selective to a specific substrate or a class of substrates. The second advantage is that during catalytic turnover, enzymes can produce ions, protons, heat, light, and/or electrons which are all measurable parameters [2].

#### 2.1.2. Antibody-Based Biosensor

Recently, antibodies (Abs) have been applied widely [20] as recognition elements in biosensors in the most rapid detection systems [21,22]. The use of antibodies as recognition elements has attracted attention [22] especially after establishing the monoclonal antibody (Mab) technology by Kohler and Milstein [23]. One of the major advantages of the antibody-based biosensor is that the immunogen (*i.e.*, analyte of interest) does not need to be purified prior to detection [21,24]. Recently, recombinant antibodies have been successfully created by genetically modified antigen binding sites (Fab fragment) of common antibodies [25].

#### 2.1.3. Aptamer-Based Biosensor

In the past three decades, research in aptamers have grown dramatically and become one of the most important biological recognition elements competing with antibodies in the development of biosensors [26,27]. Aptamers are defined as amino acid polymers or a single-stranded nucleic acid that have a high selectivity, specificity and affinity towards a target analyte ranging from small molecules to whole cells [26,28]. Generally, an *in vitro* selection method called Systematic of Ligands by Exponential enrichment (SELEX) [28] is used to isolate aptamers from large combinatorial libraries containing approximately 1015 different sequences [29].

Aptamers have the ability to bind to their specific ligands with dissociation constants in the micromolar to picomolar range [20], and can be selected for a wide range of analytes such as pathogens, toxins [30], proteins [30,31], and whole cells [32]. Some of the advantages of aptamers as biological recognition elements over antibodies include the aptamer’s long-term stability [33], their inexpensive and rapid synthesis [33,34], and their ability to be modified with labels with little effect on their binding site performance, stability, or binding characteristics [33,35]. In any case of these biological recognition elements mentioned above, they are commonly immobilized onto a solid support so that it cannot be readily removed but can still react freely with its substrate. In essence, the main advantages of an immobilized biological recognition element include easy separation from the reaction mixture and the ability to control reaction times and minimize the biological recognition elements lost in the reaction mixture [16].

### 2.2. Biosensors Categorized Based on the Type of Transducers

Biosensors can be classified into several different kinds according to the kind of transducer used. The transducer transforms the biomolecule-analyte interaction into a measurable optical [24] or electrical signal [36]. The selection of the transducer depends on the nature of the physicochemical change of the reaction that takes place at the sensing layer generated [3]. Both transducers and biological recognition elements are important for enhancing the sensitivity and detection limit of the biosensor [36].

A wide range of transducers has been developed and employed; however, the most popular methods are: (a) electrochemical; (b) optical (including colorimetric); (c) piezoelectric; and (d) magnetic [37]. Table 1 summarizes different sensing techniques with their advantages and disadvantages for the detection of a range of analytes using the most common transducers.

#### 2.2.1. Electrochemical-Based Biosensors

Recently, most of the biosensors used in the literature are mainly based on electrochemical detection [38,39]. It has been suggested that electrochemical biosensors will be responsible for a big improvement in the future of genetic testing [40]. High sensitivity, low power requirements, low cost, and relatively simple instrumentation [40,41] make electrochemical detection methods highly compatible for the development of biosensors. In general, there are three categories of electrochemical sensors that can be used to detect any changes in the electrochemical responses occurring during the reaction. These categories are defined based on the detected parameter which include amperometric (current), potentiometric (potential), and impedance (impedimetric) [42].

**Table 1:** Different examples of sensing techniques with their advantages and disadvantages.

Transducer	Technique	Advantages	Disadvantages
Electrochemical	Amperometric [43,44]	Simplicity, miniaturization, low cost	Need redox elements to enhance the current production; time consuming; sensitive to the surrounding environment
	Potentiometric [45,46]	Real-time detection; the possibility of continuous analysis on different analytes	Sensitive to the surrounding environment; time consuming; sensitive to temperature
	Impedimetric [47,48]	Simplicity and real-time detection	Sensitive to the surrounding environment; bulky devices required; require theoretical stimulation for data analysis
Optical	Surface plasmon resonance (SPR) [49,50]	Real-time detection; reliable, high sensitivity	Sensitive to the surrounding environment; surface modification as one of the main challenges; bulky optical devices required
Mechanical	Cantilever [51,52]	Real-time detection; ability to detect more than one analyte with high sensitivity	Sensitive to the surrounding environment; sensitive to temperature; bulky devices required
	Quartz crystal microbalance (QCM) [53,54]	Real-time detection; simplicity; high compatibility with point-of-care (POC) devices	Sensitive to the surrounding environment; sensitive to temperature and stress

#### 2.2.2. Optical-Based Biosensors

Optical biosensing is a vital analytic and detection technique, which has a wider application in the field of medical diagnostics [55,56] , food analysis [57,58] , environmental applications [59,60], drug discovery [61,62], and security and defense [63,64]. Optical techniques are used to detect the optical change due to the interaction between the target of interest and the biological recognition element (immobilized on the optical sensing layer), and transform the signal to a quantifiable measurement which is correlated to the analyte concentration in the sample [65]. They have many advantages, making optical detection one of the leading detection methods in the biosensor field. These advantages include low detection limit, versatility, label-free, non-destructive, and their ability to detect a wide variety of analytes or multiple analytes at the same time with fast signal monitoring and analysis [66]. Optical biosensors use either direct detection of the analyte (such as UV absorption [67], planar optical waveguide [68], fiber optics [69], surface-enhanced Raman Scattering (SERS) [70] and surface plasmon resonance (SPR) [71]), or indirect detection through optically labelled probes (such as fluorescence and chemiluminescence [72]).

#### 2.2.3. Colorimetric Biosensors

Most of the colorimetric detection techniques involve monitoring the formed colored products as a result from the reaction between the analyte of interest and biological recognition element. The colored product, which can be identified by a naked eye or the use of an optical sensing instrument, is proportional to the analyte concentration. These types of detection are classified as a label-free detection method [73].

#### 2.2.4. Mass Biosensors

One of the unique signal transduction methods in the configuration of biosensor is the fast mass transformation occurring due to the interaction between the analyte and the immobilized biological recognition element. When the mass of the crystal increases due to specific reaction or binding of an analyte to biological layer immobilized on the surface, a change in the oscillation frequency of the crystal occurs, identifying the concentration of the analyte [74]. This type of biosensors is very sensitive and can measure even small changes of the interacting molecules weight on the crystal surface [75].

#### 2.2.5. Magnetic Sensors

The use of magnetic sensing that relies on the use of micro/nanoparticles labelling has increased in recent years. Bio-molecules have virtually no magnetic properties; therefore, the addition of magnetic micro/nanobeads to bio-molecular samples could be used to separate, and quantify a known analyte within a given sample [76]. A number of magnetic platforms have been used to detect a number of disease targets such as proteins [77], cancer cells [78], enzymes and pathogens.

## 3. Microfluidics

**Figure 3:**
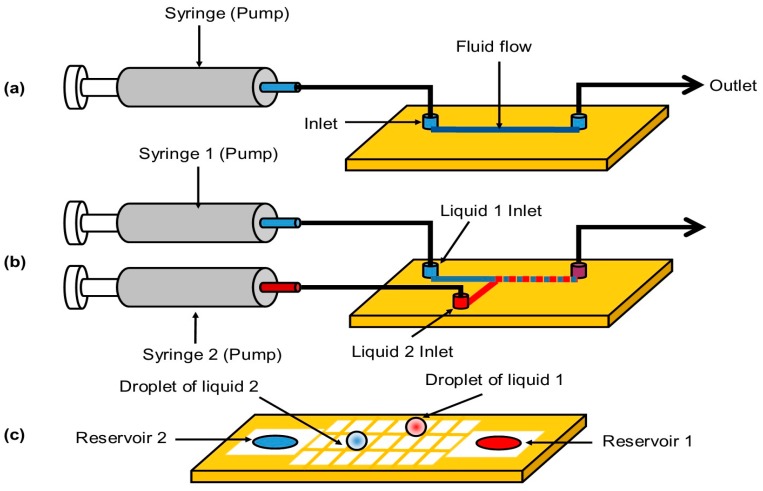
Schematic of the three microfluidic systems (**a**) continuous; (**b**) drop-based; and (**c**) digital.

Numerous microfluidic devices have been developed for different biological and chemical applications [79]. Conventional microfluidic systems are based on the continuous flow regimes in micron-sized channels (Figure 3a). These micro-channels are fabricated mainly using soft-lithography methods [80]. To reduce the sample consumption, and also create isolated reaction sites, droplet-based microfluidic systems have been developed [81]. The first generation of the droplet-based microfluidic systems utilized the continuous stream of two or more fluid mainly intersected at a T-junction to create discrete droplets which are isolated from each other using an immiscible fluid (Figure 3b) [82]. To further decrease the volume consumption, a new generation of droplet-based microfluidic systems, called digital microfluidic (DMF), was introduced in early 2000s [83]. Rather than having continuous flow of droplets in the micro-channels, DMF systems create droplets on an array of electrostatically actuated electrodes (Figure 3c). The most commonly used actuation mechanism for moving the droplets on the array of electrodes is electrowetting-on-dielectric (EWOD) technique which is based on changing the interfacial properties of the liquid using an electric field [84]. EWOD method provides higher localization compared to the other methods. In addition to lower power consumption, the EWOD-based DMF systems have many advantages over the continuous microfluidic systems such as lower power consumption and scalability [85]. These intrinsic characteristic of these systems makes them a very suitable choice for implementing additional sensing modules. In particular, in recent years, these systems have been used for numerous biosensing applications as they allow for high throughput parallel processing of multiple samples on the same chip. Table 2 summarizes the operating and actuation methods, advantages and disadvantages of the three different types of microfluidics.

**Table 2:** The comparison between the three types of microfluidics.

	Continuous-Flow Microfluidics	Droplet-Based Microfluidics	Digital Microfluidics
Operating Method	Motion of continuous fluid in micro-channels	Motion of droplets in micro-channels using streams of immiscible fluids	Motion of discrete droplets on an array of planar electrodes
Flow Actuation	Mechanical (syringe) pumps, Pneumatic pressure, Electrokinetic	Mechanical (syringe) pumps, Pneumatic pressure	Electrowetting On Dielectric, Dielectrophoresis
Advantages	Ease of fabrication and operation, suitable for applications that require a continuous flow with relatively high sampling volume, and being compatible with most of current screening and sensing mechanisms	Ease of fabrication and operation, suitable for a applications that require isolated reaction sites to avoid cross contamination	Lower sample consumption, scalability, better localization, reconfigurability, and portability
Disadvantages	High sample volume consumption compared to other microfluidic systems, possible contamination, and not being scalable due to fabrication and physical limitations	No control over individual droplets, challenging to create droplets of different sizes using the same setup, and challenging to implement stable gas-liquid systems	Complicated fabrication procedure, and bio-adsorption and evaporation

Fabrication and designing of microfluidics platforms for biosensors need careful addressing and considerations such as: dimensions, materials, and the method used for fabrication to improve the biocompatibility and wettability of the fabricated device [86]. Glass and silicon are well known to be the most common materials used in fabricating and designing microfluidic platforms. The low cost of polymer materials and their manufacturing has made them one of the popular materials used in the fabrication and designing of microfluidic devices recently [87]. The excellent chemical, physical and mechanical properties of polymers (e.g., polymethylmethacrylate (PMMA), and polydimethylsiloxane (PDMS)) have increased the biocompatibility of using them in the fabrication and designing of microfluidic devices [88]. Examples of some of the different fabrication methods and materials used to fabricate microfluidic devices for biosensing applications are summarized in Table 3.

**Table 3:** Examples of some of the different fabrication methods and materials used to fabricate microfluidic devices for biosensing applications.

Fabrication Method	Fabrication Material	Advantages	Disadvantages
Photolithography [89]	PDMS	Portability	Low throughput
		Cost-effective and high automation	
		High sensitivity	
Soft lithography [90]	PDMS	Real-time detection	Requiring high sample concentration
		Portable	
		Disposable	
		Cost-effective	
Nano-imprinting [91]	PMMA	Cost-effective	Expensive Low throughput
		High sensitivity	

## 4. Integration of Microfluidics with Biosensor Technology

Recently, a significant demand and effort in merging biosensors into lab-on-chip (LOC) technology using microfluidics systems has been demonstrated [92,93] which add numerous benefits to the biosensor technology [94]. The integration of biosensors with microfluidic systems offers an integrated and miniaturized alternative to the traditional repetitive laboratory methods [95,96], as it offers significant reduction in sample, reagent, energy consumption [97,98], and waste production [99]. Moreover, the microfluidic biosensors can decrease the cost, and increase the specificity and detection sensitivity limit compared to the regular detection methods.

Due to the small size of micro-systems, a single microfluidic biosensor can perform full analysis [100] including continuous sampling, sample separation and mixing [101], and pre-concentration and treatment [102]. Furthermore, these microfluidic biosensors offer enhanced analytical performance, high throughput, real-time detection, fast reaction rates and portability [103,104], making detection adaptable to point of care (POC) applications [105]. Overall, the integration of biosensors with the microfluidic systems creates a powerful analytical tool that will be an advanced step towards the home-testing approach which will benefit both developing and developed countries.

In the next section, examples of microfluidics based biosensors will be explained based on the different kinds of microfluidics and the biological recognition element used as the sensing layer in the biosensor. Table 4 shows a comparison between some of the common biological recognition elements used in microfluidic systems for biosensing applications with their advantages and disadvantages.

**Table 4:** The common biological recognition elements used in microfluidic devices.

Biological Recognition Element	Advantages	Disadvantages
Enzymes [95,97]	High sensitivity	Possibility of losing their activity upon immobilization
	High selectivity towards their targets	
	Suitable for oxidation reduction reactions	Most suitable for small analytes, e.g., glucose, urea and lactate
Antibodies [106,107]	Rapid analysis for direct immunoassays	Requiring labeling for indirect immune assays which can result in the increase cost and time required for analysis
	Suitable for bioaffinity interaction e.g., antibody-antigen interaction	Not suitable for detection of small targets using direct and sandwich immunoassays
	Suitable for the detection of large targets e.g., bacteria and pathogens	Not suitable for oxidation reduction reactions
Aptamers [108,109]	Highly sensitive and selective	Higher toxicity than antibodies
	Suitable for the detection of a wide range of analytes	Faster excretion due to their small size
	Long-term stability, inexpensive and rapid synthesis	Weaker binding to analytes
	Flexibility to be modified with labels without losing their performance or binding properties	

### 4.1. Continuous Microfluidic-Based Biosensor

In the last two decades, the continuous microfluidic system has improved. The huge advancement in microfluidic research has increased the study of biological systems ranging from molecules to small multicellular organisms. Microfluidics has added a great ability to sensing devises over conventional methods as it can sense small volumes of analytes, resulting in reducing reagent and energy consumption, less waste, reducing the cost and the integration of chemical and biological process on a single platform [104]. Recently, continuous microfluidic has been widely used in unique different applications such as: (a) chemical and system biology [106,107]; (b) biological screening and drug discovery [96]; (c) clinical diagnostic [107]; (d) point-of-care devices for environmental and (e) biomedical applications [109]. In the following subsections, examples of continuous microfluidics-based biosensors using different biological recognition element are illustrated.

#### 4.1.1. Enzyme-Based

**Figure 4:**
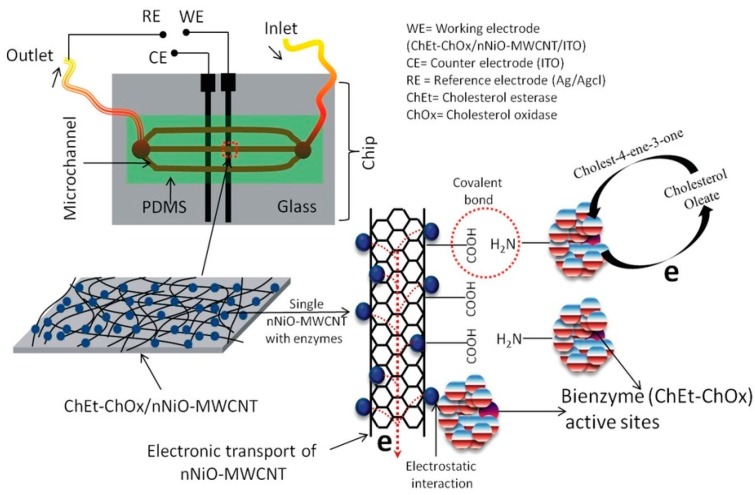
Schematic of the bienzyme functionalized nanocomposite microfluidic-based biosensor developed for the detection of cholesterol (Reprinted with permission from Scientific Reports) [110].

A few examples on the application of the biosensor technology and its integration with the continuous microfluidic platforms have been reported and demonstrated in the past. For instance, an example for the integration of bienzyme functionalized nanocomposite with a microfluidic biosensor was demonstrated by Ali *et al.* [110] (see Figure 4). In this work, they have illustrated successful fabrication and integration of a novel microfluidics biochip using a multi-walled carbon nanotubes (MWCNTs) and nickel oxide nanoparticles (nNiO). Photolithographic technique was used to integrate the nanocomposite with the polydimethylsiloxane (PDMS) microchannels. The surface of the integrated nanocomposite-microchannels was functionalized with two enzymes including cholesterol oxidase (ChOx) and cholesterol esterase (ChEt). The final structure was then characterized using X-ray photoelectron spectroscopy (XPS) and scanning electron microscopy techniques. The fabricated chip was used to measure the chronoamperometric change in the presence of different concentrations of cholesterol oleate (0.25–12.93 mM). The results showed a linear relation between the chronoamperometric change and the cholesterol oleate concentrations. The novel integrated system provided high reproducibility, selectivity and excellent sensitivity of 2.2 mA/mM/cm^2^.

#### 4.1.2. Antibody-Based

**Figure 5:**
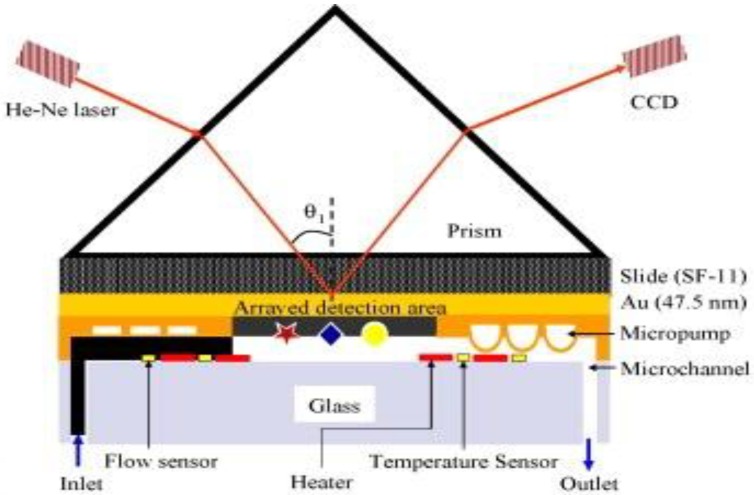
Illustration of 2D surface plasmon resonance (SPR) imaging integrated with a microfluidic platform for the detection of microarray immunoassay samples (Reprinted with permission from Elsevier) [111].

An example for the integration of a continuous microfluidic device and biosensor using antibodies as the biological recognition element is demonstrated by Lee *et al.* [111] for the detection of breast cancer using the SPRi method. They have managed to develop an automated lab-on-a-chip microfluidic biosensor with multiple channels for the detection of a microarray samples (see Figure 5). Since SPR is a temperature dependent technique, they used a temperature control unit consisting of micro- heaters and temperature sensors to avoid temperature change and variation in the sensing area during the experiment. Regular self-assembled monolayer (SAM) was used to immobilize the anti-rabbit IgG on the modified gold surface. They achieved high specificity and selectivity by delivering and accurate amounts of IgG solution to the detection are using micropumps/valves. The results showed that the fabricated microfluidic platform, which used SPR phase imaging successfully detected the presence of only IgG in the samples. The novel integrated system provided a high selectivity and excellent sensitivity with a detection limit of 1 × 10^−4^ mg/mL (0.67 nM) which provide a highly sensitive and selective, rapid and low-cost sensing platform for the detection of biomedical samples and protein-protein interactions.

#### 4.1.3. Aptamer-Based

**Figure 6:**
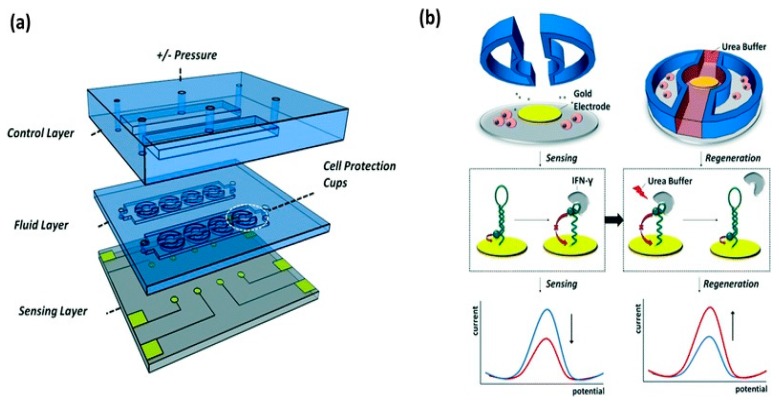
(**a**) Schematic of the whole layout of the aptamer microfluidic-based biosensor; (**b**) the steps taken for the development of the biosensor and the principle of detection of the sensing of cytokine. The regeneration of cytokine is explained in the lower panel by showing the square wave signals during sensing (left) and regeneration steps (right) (Reprinted with permission from Royal Society of Chemistry) [113].

There are several advantages that make aptamers a preferable biological recognition element over antibodies. These advantages include simplicity in the structure of aptamers and the ability to design them in a way to yield an optical or electrical signal (upon binding with the target) without the need for secondary labeling and washing steps, reducing the cost and time [108,112]. An excellent example for an on-chip aptamer-based sensor was developed by Zhou *et al.* [113] for the continuous detection of living cells (see Figure 6). Their microdevice was fabricated on glass substrate with gold electrodes and two polydimetylsiloxane (PDMS) layers as shown in Figure 6a. The two PDMS layers were designed so that the first layer contains microchannels and semi-circular microcups, and the second layer was mainly used for pneumatic control. The microcups were raised during the protein secretion by cells which increased protein diffusion toward the sensing area. The microcups are then lowered during the physical separation of cells from the sensing electrode area (see Figure 6b). Square wave voltammetry (SWV) measurements were used to confirm the interaction between the cell-secreted protein (IFN-γ) and the specific aptamer. The results showed a decrease in the redox signal which is proportional to the analyte concentration in the sample. The novel integrated system provided a regenerative aptamer microfluidic-based biosensor for the continuous monitoring of proteins secreted by cells with a detection limit of (5 ng·mL^−1^).

### 4.2. Droplet Microfluidic-Based Biosensor

Droplet-based microfluidics is one of the most significant systems to be integrated with the biosensor technology. Recently, droplet-based microfluidics has extensively been used as a new platform for a wide range of applications including environmental, biomedical, security and defense with a better portability, and low energy [114]. In the following subsections, examples of droplet microfluidics-based biosensors using different biological recognition element are illustrated.

#### 4.2.1. Enzyme-Based

**Figure 7:**
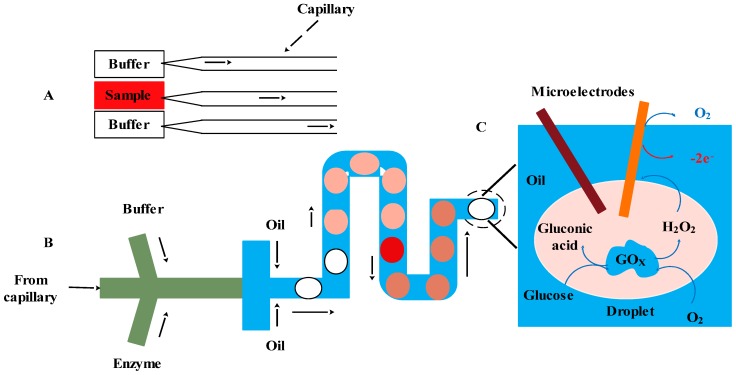
Schematic of (**A**) a droplet-based electrochemical biosensor for monitoring glucose using glucose oxidase; (**B**) the droplet-based electrochemical sensor; and (**C**) the monitoring of glucose in droplet (**C**).

An example of a droplet-based microfluidic electrochemical sensor using Pt-black microelectrode and enzymes for the detection of glucose was demonstrated by Gu *et al.* [115] (see Figure 7). Electrochemical impedance spectroscopy (EIS) and cyclic voltammetry (CV) were used to measure the change in the electrochemical current due to the oxidation of β-d-glucose, resulting in the byproduct of H_2_O_2_. The novel integrated microfluidic based electrochemical biosensor system provides a highly sensitive and low-cost glucose sensor with linear response up to 43.5 mM.

#### 4.2.2. Antibody-Based

**Figure 8:**
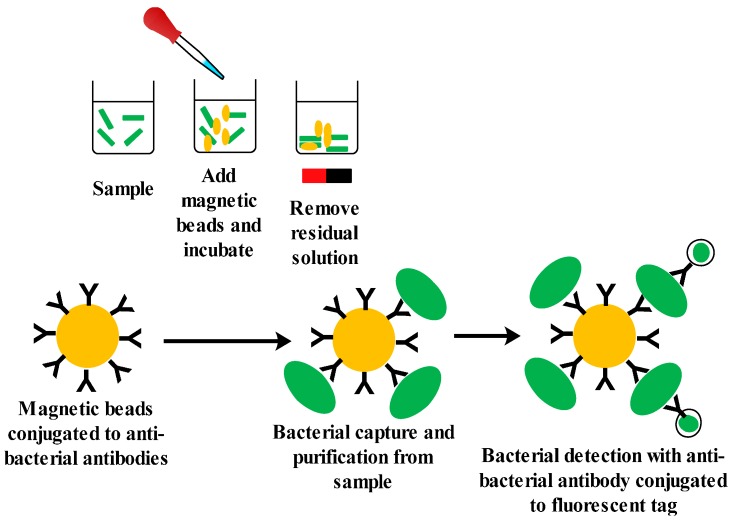
Schematic of the droplet-based electrochemical biosensor for the detection of *E. coli* using magnetic beads conjugated with antibodies as the biological recognition element.

A novel all-in-one droplet-based microfluidic biosensor (Scandrop) has recently been developed by Golberg *et al.* [116] using magnetic beads conjugated with antibodies as the biological recognition element which are specific for the capture and the detection of bacteria *E. coli* in drinking water. First anti-*E. coli* antibodies conjugated magnetic beads were used to selectively capture and isolate *E. coli* from the contaminated water. The isolated bacteria were then co-encapsulated with fluorescently-labeled anti-*E. coli* antibodies in pico-liter droplets and were analyzed using an automated fluorescence microscope. The detection process required only 8 h of sample collection, pre-concentration, capturing and detection (see Figure 8). The system has shown a great potential towards the development of droplet-based microfluidic biosensor for monitoring of pathogens in drinking water compared to the conventional methods requiring 2–4 days for the detection of pathogens.

#### 4.2.3. Aptamer-Based

**Figure 9:**
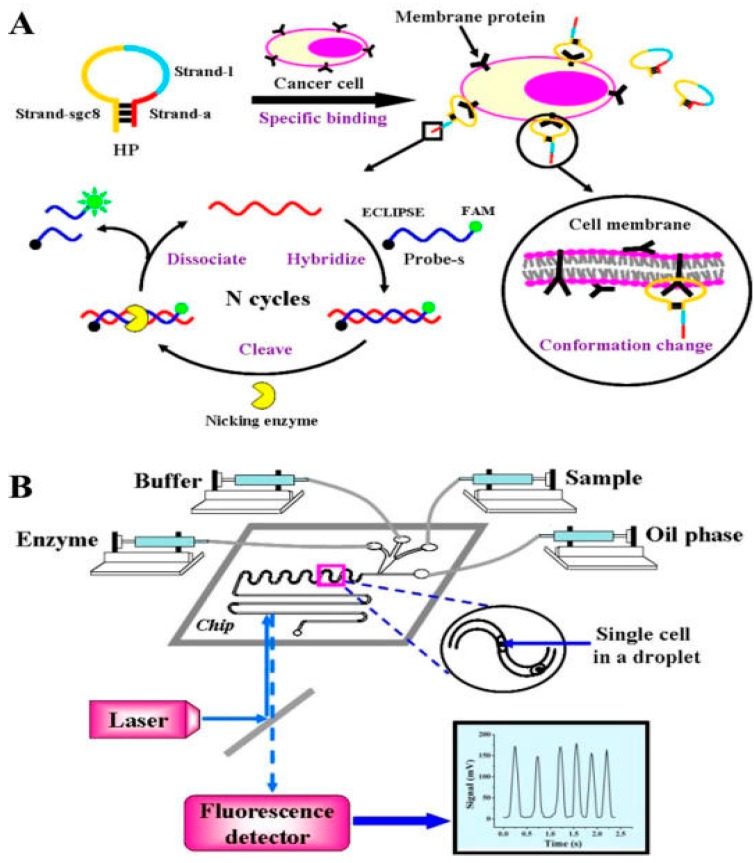
(**A**) Schematic of the sensitive detection of the membrane protein on single living cells using aptamer and nicking enzyme as the biological sensing element; (**B**) Schematic of the whole droplet-based microfluidic system (Reprinted with permission from American Chemical Society) [118].

Recently, a “finger-powered” bead-in-droplet microfluidic system was developed using aptamer-beacons to detect the inflammatory cytokine interferon gamma. Droplets containing aptamer-functionalized microbeads were generated by a finger push-and-release sequence. When interferon gamma was present within the droplets, the fluorescence intensity of the aptamer-beacons was detectable [117]. In another example, the detection of a membrane protein PTK7 on single living cells was demonstrated using aptamers in droplet-based microfluidics. The droplets served as independent microreactors where a fluorescence amplification reaction was taken place between the aptamer and a nicking enzyme, allowing for very sensitive, high throughput detection [118] (see Figure 9).

### 4.3. Digital Microfluidic-Based Biosensor

Digital microfluidics (DMF), as another approach used for the manipulation of liquid samples as microdroplets, has offered reconfigurability and flexibility and limited contamination during sample preparation and analysis. This type of platform is compatible with a wide range of detection methods (such as optical and electrochemical detection [83]) and eliminates the use of conventional pumps, valves or channels, unlike the continuous microfluidics [119]. Integrating of biosensors into the DMF platforms has enhanced the functionality of different fluidic operations (e.g., transport, mixing, splitting and separation) developed for DMF platforms so far [120,121] . In the following subsections, examples of digital microfluidics-based biosensors using different biological recognition element are illustrated.

#### 4.3.1. Enzyme-Based

**Figure 10:**
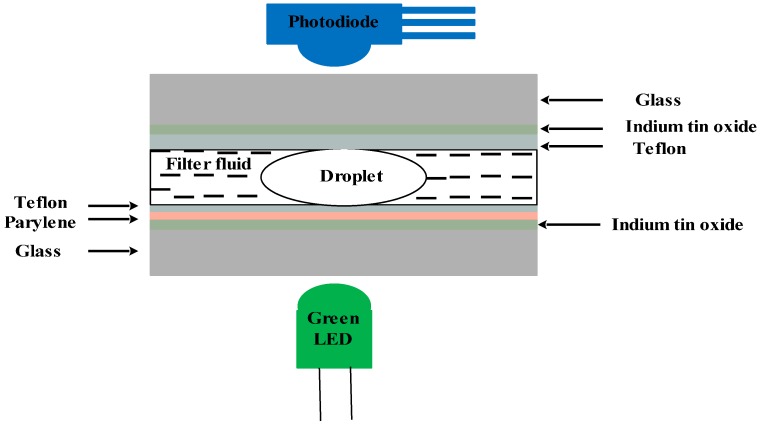
Schematic of the digital microfluidic-based optical biosensor for the rapid detection of glucose in a droplet sample.

The integration of optical absorbance measurements system with digital microfluidics for the detection of body metabolites using glucose oxidase as the biological recognition element has been reported by Srinivasan *et al.* [122] (see Figure 10). The developed devise was also tested towards the detection of glucose in the droplet sample using a colorimetric enzyme-kinetic assay. For this purpose, they integrated a colorimetric enzyme-kinetic assay into the digital microfluidic platform, and hence developed an optical DMF-based biosensor for the rapid detection of glucose in less than 40 s with a linear response in the range of 25 mg/dl to 300 mg/dl, with less than 5% linearity deviation from the high limit. The developed system was successfully tested for the detection of glutamate, pyruvate, and lactate.

#### 4.3.2. Antibody-Based

**Figure 11:**
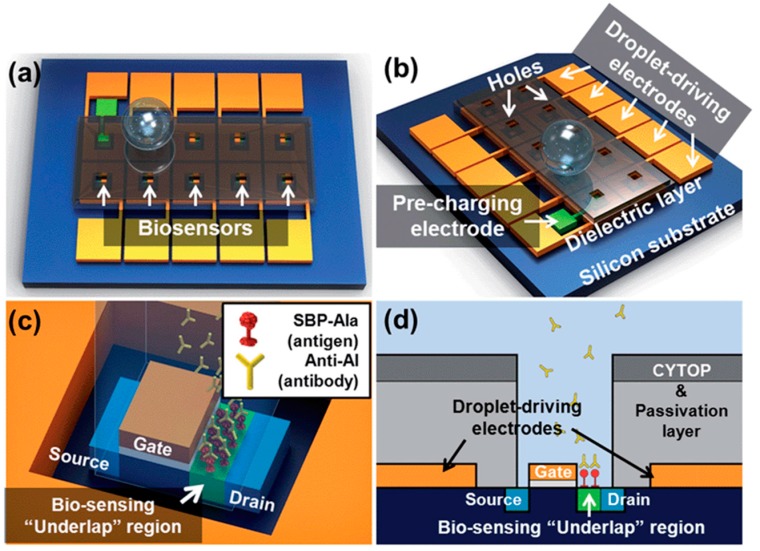
Schematics of the developed field effect transistor (FET) digital microfluidic biosensor: (**a**) a top-view; (**b**) a side-view; (**c**) an enlarged view of the FET digital microfluidic biosensor and (**d**) a cross-sectional view of the FET digital microfluidic biosensor (Reprinted with permission from Royal Society of Chemistry) [123].

The integration of field effect transistor (FET) biosensor into the digital microfluidic technology using antibodies as the biological recognition element was demonstrated by Choi *et al.* [123]. They developed a new digital microfluidic FET-based biosensor for the real time detection of avian influenza antibody (anti-AI). The device works based on the measurement of the current drained from the FET biosensor without the need for using labels (see Figure 11). The electrowetting-on-dielectric method (EWOD) was used to deliver the droplet containing the analyte anti-AI from the inlet to the sensing area. The results showed a decrease in the drain current upon the binding of the target antigen and the specific anti-AI antibodies. The system has shown a great potential towards the detection of anti-AI with a detection limit of 0.5 pg·mL^−1^ (6.67 fM).The integration of digital microfluidics with the FET-based sensor using antibodies as the biological recognition element has shown a great potential towards biomolecules transportation and detection without the need of pumps, bulky transduces or small fluidic channels.

#### 4.3.3. Aptamer-Based

Aptamers have been used in conjunction with antibodies in digital microfluidic biosensors. A droplet with fluorescently labeled IgE aptamer and magnetic nanoparticles coated with anti IgE was merged with a second droplet containing unlabeled IgE. The mixture was brought to a permanent magnet to hold the magnetic beads. The droplets of a washing buffer were passed over the beads to remove unbound IgE from the magnetic beads. The assay had a limit of detection (LOD) of 150 nM of IgE [121].

## 5. Conclusions

Standard methods for identification and detection of specific targets are expensive, time consuming, and suffer from the lack of portability. The integration of microfluidics and biosensors provides a powerful tool to replace the bulky traditional instruments with the ability to merge chemical and biological components into a single platform. Biosensors are considered to be a powerful analytical tools and are potentially useful for a wide range of applications ranging from drug discovery, to medical diagnostics, to food safety, to agricultural and environmental monitoring, and to security and defense [1]. A biosensor can be defined as an analytical device [1,2] that combines a biological sensitive recognition element [3] immobilized on a physicochemical transducer connected to a detector to identify the presence of one or more specific analytes [4], their concentrations and kinetics in a sample. The specificity and selectivity of the biosensor is mainly based on the affinity properties of the biological recognition element. The signal originating from the interaction between the analyte of interest and the biological recognition element is then transformed by a transducer to an optical or electrical readout [5,6]. Microfluidics technology, on the other hand, provides great ability to perform and analyze complex operations including chemical and system biology [106,107], biological screening and drug discovery [61,62], clinical diagnostic [107], detection of different toxins [124], and inexpensive point-of-care devices for environmental, biomedical applications in developing and developed countries [109]. The advances made in the soft lithography techniques have even made the design of new microfluidic platforms much easier and faster. The use of microfluidic devices has provided an opportunity to perform laboratory processes [95,96] using small volumes of analytes, resulting in reduction of reagents, lower energy consumption [97], real-time detection, and simultaneous analysis of different and multiple analytes on a single platform (especially required for point-of-care testing) [98,99]. This paper presents a review of the advances and successes made in the fields of biosensors and microfluidics, and especially microfluidic-based biosensors in different applications. Different types of biosensors (with different types of biological recognition elements including enzymes, antibodies, and aptamers) and microfluidic systems (*i.e.*, continuous, droplet-based, and digital microfluidics) were presented. The most important part of this review includes examples of integrated biosensors into microfluidic platforms, illustrating the versatility of such as integration for emerging areas of biological engineering, biomedical studies, point-of-care diagnostics, and environmental monitoring.

## References

[1] Lindholm-Sethson B., Nyström J., Geladi P., Koeppe R., Nelson A., Whitehouse C. (2003). Are biosensor arrays in one membrane possible? A combination of multifrequency impedance measurements and chemometrics. Anal. Bioanal. Chem..

[2] Subrahmanyam S., Piletsky S.A., Turner A.P.F. (2002). Application of Natural Receptors in Sensors and Assays. Anal. Chem..

[3] Mohanty S.P., Kougianos E. (2006). Biosensors: A tutorial review. IEEE Potentials.

[4] Rasooly A., Jacobson J. (2006). Development of biosensors for cancer clinical testing. Biosens. Bioelectron..

[5] Holford T.R.J., Davis F., Higson S.P.J. (2012). Recent trends in antibody based sensors. Biosens. Bioelectron..

[6] Turner A.P.F. (2013). Biosensors: Sense and sensibility. Chem. Soc. Rev..

[7] Jain P., Chakma B., Patra S., Goswami P. (2014). Potential biomarkers and their applications for rapid and reliable detection of malaria. Biomed Res. Int..

[8] Wang J., Ren L., Li L., Liu W., Zhou J., Yu W., Tong D., Chen S. (2009). Microfluidics: A new cosset for neurobiology. Lab Chip.

[9] Henares T.G., Mizutani F., Hisamoto H. (2008). Current development in microfluidic immunosensing chip. Anal. Chim. Acta.

[10] Paper R. (2008). Electrochemical Biosensors—Sensor Principles and Architectures. Sensors.

[11] The D.R., Toth K., Durst R.A., Wilson G.S. (2001). Technical report Electrochemical biosensors: Recommended definitions and classification. Biosens. Bioelectron..

[12] Ruan C., Zeng K., Varghese O.K., Grimes C.A. (2004). A magnetoelastic bioaffinity-based sensor for avidin. Biosens. Bioelectron..

[13] Long F., Zhu A., Shi H. (2013). Recent advances in optical biosensors for environmental monitoring and early warning. Sensors.

[14] Cai H., Zhou C. SAW based mass-loading biosensor for DNA detection. Proceedings of the 2013 IEEE International Conference of Electron Devices and Solid-State Circuits (EDSSC).

[15] Kissinger P.T. (2005). Biosensors-a perspective. Biosens. Bioelectron..

[16] Datta S., Christena L.R., Rajaram Y.R.S. (2012). Enzyme immobilization: An overview on techniques and support materials. 3 Biotech.

[17] Santano E., Pinto C., Macías P. (2002). Xenobiotic oxidation by hydroperoxidase activity of lipoxygenase immobilized by adsorption on controlled pore glass. Enzyme Microb. Technol..

[18] Hilvert D. (2000). Critical anallysis of antibody catalysis. Annu. Rev. Biochem..

[19] Clark L., Lyons C. (1962). Electrode systems for continous monitoring in cardiovascular surgery. Ann. N. Y. Acad. Sci..

[20] Jayasena S.D. (1999). Aptamers: An Emerging Class of Molecules That Rival Antibodies in Diagnostics. Clin. Chem..

[21] Saerens D., Huang L., Bonroy K., Muyldermans S. (2008). Antibody Fragments as Probe in Biosensor Development. Sensors.

[22] Yalow R. (1959). Assay of plasma insulin in human subjects by immunological methods. Nature.

[23] Kohler G., Milstein C. (1975). Continuous cultures of fused cells secreting antibody of predefined specificity. Nature.

[24] Cheng A.K.H., Ge B., Yu H.-Z. (2007). Aptamer-based biosensors for label-free voltammetric detection of lysozyme. Anal. Chem..

[25] Emanuel P.A., Dang J., Gebhardt J.S., Aldrich J., Garber E.A.E., Kulaga H., Stopa P., Valdes J.J., Dion-schultz A. (2000). Recombinant antibodies: A new reagent for biological agent detection. Biosens. Bioelectron..

[26] Nimjee S.M., Rusconi C.P., Sullenger B.A. (2005). Aptamers: An emerging class of therapeutics. Annu. Rev. Med..

[27] McKeague M., Velu R., Hill K., Bardóczy V., Mészáros T., DeRosa M.C. (2014). Selection and characterization of a novel DNA aptamer for label-free fluorescence biosensing of ochratoxin A. Toxins.

[28] Ellington A., Szostak J. (1990). *In vitro* selection of RNA molecules that bind specific ligands. Nature.

[29] Cruz-Toledo J., McKeague M., Zhang X., Giamberardino A., McConnell E., Francis T., DeRosa M.C., Dumontier M. (2012). Aptamer Base: A collaborative knowledge base to describe aptamers and SELEX experiments. Database.

[30] McKeague M., Bradley C.R., De Girolamo A., Visconti A., Miller J.D., Derosa M.C. (2010). Screening and initial binding assessment of fumonisin b(1) aptamers. Int. J. Mol. Sci..

[31] Lin H., Zhang W., Jia S., Guan Z., Yang C.J., Zhu Z. (2014). Microfluidic approaches to rapid and efficient aptamer selection. Biomicrofluidics.

[32] Bayrac A.T., Sefah K., Parekh P., Bayrac C., Gulbakan B., Oktem H.A., Tan W. (2011). *In vitro* Selection of DNA Aptamers to Glioblastoma Multiforme. ACS Chem. Neurosci..

[33] Ciobanu M., Cliffel D. (2008). Glucose and Lactate Biosensors for Scanning Electrochemical Microscopy Imaging of Single Live Cells. Anal. Chem..

[34] Ramalingam D., Duclair S., Datta S.A.K., Ellington A., Rein A., Prasad V.R. (2011). RNA aptamers directed to human immunodeficiency virus type 1 Gag polyprotein bind to the matrix and nucleocapsid domains and inhibit virus production. J. Virol..

[35] Campanella L., Favero G., Persi L., Sammartino M., Tomassetti M., Visco G. (2001). Organic phase enzyme electrodes: Applications and theoretical studies. Anal. Chim. Acta.

[36] Singh M., Kathuroju P.K., Jampana N. (2009). Polypyrrole based amperometric glucose biosensors. Sens. Actuators B Chem..

[37] Search H., Journals C., Contact A., Iopscience M., Address I.P. (1997). Biosensors: Recent advances. Reports Prog. Phys..

[38] Bo S., Pijanowska D., Olthuis W., Bergveld P. (2001). A flow-through amperometric sensor based on dialysis tubing and free enzyme reactors. Biosens. Bioelectron. Bioelectron..

[39] Radoi A., Compagnone D. (2009). Recent advances in NADH electrochemical sensing design. Bioelectrochemistry.

[40] Wang J. (2002). Electrochemical nucleic acid biosensors. Anal. Chim. Acta.

[41] D’Orazio P. (2003). Biosensors in clinical chemistry. Clin. Chim. Acta.

[42] Warsinke A., Benkert A., Scheller F.W. (2000). Electrochemical immunoassays. Fresenius. J. Anal. Chem..

[43] Shi W., Ma Z. (2011). A novel label-free amperometric immunosensor for carcinoembryonic antigen based on redox membrane. Biosens. Bioelectron..

[44] Qiu J.D., Huang H., Liang R.P. (2011). Biocompatible and label-free amperometric immunosensor for hepatitis B surface antigen using a sensing film composed of poly(allylamine)-branched ferrocene and gold nanoparticles. Microchim. Acta.

[45] Zelada-Guillén G.A., Tweed-Kent A., Niemann M., Göringer H.U., Riu J., Rius F.X. (2013). Ultrasensitive and real-time detection of proteins in blood using a potentiometric carbon-nanotube aptasensor. Biosens. Bioelectron..

[46] Koncki R. (2007). Recent developments in potentiometric biosensors for biomedical analysis. Anal. Chim. Acta.

[47] Danielsa J.S. (2008). Label-Free Impedance Biosensors: Opportunities and Challenges. Electroanalysis.

[48] Guo X., Kulkarni A., Doepke A., Halsall H.B., Iyer S., Heineman W.R. (2012). Carbohydrate-Based Label-Free Detection of Escherichia coli ORN 178 Using Electrochemical Impedance Spectroscopy. Anal. Chem..

[49] Guo X. (2012). Surface plasmon resonance based biosensor technique: A review. J. Biophotonics.

[50] Tawil N., Sacher E., Mandeville R., Meunier M. (2012). Surface plasmon resonance detection of *E. coli* and methicillin-resistant S. aureus using bacteriophages. Biosens. Bioelectron..

[51] Tamayo J., Kosaka P.M., Ruz J.J., San Paulo Á., Calleja M. (2013). Biosensors based on nanomechanical systems. Chem. Soc. Rev..

[52] Liu Y., Schweizer L.M., Wang W., Reuben R.L., Schweizer M., Shu W. (2013). Label-free and real-time monitoring of yeast cell growth by the bending of polymer microcantilever biosensors. Sens. Actuators B Chem..

[53] Lu C.H., Zhang Y., Tang S.F., Fang Z.B., Yang H.H., Chen X., Chen G.N. (2012). Sensing HIV related protein using epitope imprinted hydrophilic polymer coated quartz crystal microbalance. Biosens. Bioelectron..

[54] Cheng C.I., Chang Y.-P., Chu Y.-H. (2012). Biomolecular interactions and tools for their recognition: Focus on the quartz crystal microbalance and its diverse surface chemistries and applications. Chem. Soc. Rev..

[55] Bottazzi B., Fornasari L., Frangolho A., Giudicatti S., Mantovani A., Marabelli F., Marchesini G., Pellacani P., Therisod R., Valsesia A. (2014). Multiplexed label-free optical biosensor for medical diagnostics. J. Biomed. Opt..

[56] Pal S., Yadav A.R., Lifson M.A., Baker J.E., Fauchet P.M., Miller B.L. (2013). Selective virus detection in complex sample matrices with photonic crystal optical cavities. Biosens. Bioelectron..

[57] McGrath T.F., Andersson K., Campbell K., Fodey T.L., Elliott C.T. (2013). Development of a rapid low cost fluorescent biosensor for the detection of food contaminants. Biosens. Bioelectron..

[58] Xu X., Liu X., Li Y., Ying Y. (2013). A simple and rapid optical biosensor for detection of aflatoxin B1 based on competitive dispersion of gold nanorods. Biosens. Bioelectron..

[59] Liu S., Zheng Z., Li X. (2013). Advances in pesticide biosensors: Current status, challenges, and future perspectives. Anal. Bioanal. Chem..

[60] Zhong Z., Fritzsche M., Pieper S.B., Wood T.K., Lear K.L., Dandy D.S., Reardon K.F. (2011). Fiber optic monooxygenase biosensor for toluene concentration measurement in aqueous samples. Biosens. Bioelectron..

[61] Fan X., White I.M., Shopova S.I., Zhu H., Suter J.D., Sun Y. (2008). Sensitive optical biosensors for unlabeled targets: A review. Anal. Chim. Acta.

[62] Wartchow C.A., Podlaski F., Li S., Rowan K., Zhang X., Mark D., Huang K.-S. (2011). Biosensor-based small molecule fragment screening with biolayer interferometry. J. Comput. Aided. Mol. Des..

[63] Gooding J.J. (2006). Biosensor technology for detecting biological warfare agents: Recent progress and future trends. Anal. Chim. Acta.

[64] Lim D.V., Simpson J.M., Kearns E.A., Kramer M.F. (2005). Current and developing technologies for monitoring agents of bioterrorism and biowarfare. Clin. Microbiol. Rev..

[65] Hosseini S., Ibrahim F., Djordjevic I., Koole L.H. (2014). Recent advances in surface functionalization techniques on polymethacrylate materials for optical biosensor applications. Analyst.

[66] Brogan K.L., Walt D.R. (2005). Optical fiber-based sensors: Application to chemical biology. Curr. Opin. Chem. Biol..

[67] Ohlsson P.D., Ordeig O., Mogensen K.B., Kutter J.P. (2009). Electrophoresis microchip with integrated waveguides for simultaneous native UV fluorescence and absorbance detection. Electrophoresis.

[68] Kozma P., Kehl F., Ehrentreich-Förster E., Stamm C., Bier F.F. (2014). Integrated planar optical waveguide interferometer biosensors: A comparative review. Biosens. Bioelectron..

[69] Sciacca B., Monro T.M. (2014). Dip biosensor based on localized surface plasmon resonance at the tip of an optical fiber. Langmuir.

[70] Driscoll A.J., Harpster M.H., Johnson P.A. (2013). The development of surface-enhanced Raman scattering as a detection modality for portable in vitro diagnostics: Progress and challenges. Phys. Chem. Chem. Phys..

[71] Online V.A., Citartan M., Gopinath S.C.B., Tominaga J., Tang T. (2013). Label-free methods of reporting biomolecular interactions by optical biosensors. Analyst.

[72] Gabig-Ciminska M. (2006). Developing nucleic acid-based electrical detection systems. Microb. Cell Fact..

[73] Song Y., Wei W., Qu X. (2011). Colorimetric biosensing using smart materials. Adv. Mater..

[74] Leonard P., Hearty S., Brennan J., Dunne L., Quinn J., Chakraborty T., O’Kennedy R. (2003). Advances in biosensors for detection of pathogens in food and water. Enzyme Microb. Technol..

[75] Lucklum R., Hauptmann P. (2005). Acoustic microsensors—The challenge behind microgravimetry. Anal. Bioanal. Chem..

[76] Haun J.B., Yoon T.-J., Lee H., Weissleder R. (2010). Magnetic nanoparticle biosensors. Wiley Interdiscip. Rev. Nanomed. Nanobiotechnol..

[77] Li F., Kosel J. (2012). A Magnetic Method to Concentrate and Trap Biological Targets. IEEE Trans. Magn..

[78] Bi S., Cui Y., Dong Y., Zhang N. (2014). Target-induced self-assembly of DNA nanomachine on magnetic particle for multi-amplified biosensing of nucleic acid, protein, and cancer cell. Biosens. Bioelectron..

[79] Sackmann E.K., Fulton A.L., Beebe D.J. (2014). The present and future role of microfluidics in biomedical research. Nature.

[80] Xia Y., Whitesides G.M. (1998). Soft Lithography. Annu. Rev. Mater. Sci..

[81] Brouzes E., Medkova M., Savenelli N., Marran D., Twardowski M., Hutchison J.B., Rothberg J.M., Link D.R., Perrimon N., Samuels M.L. (2009). Droplet microfluidic technology for single-cell high-throughput screening. Proc. Natl. Acad. Sci. USA.

[82] Garstecki P., Fuerstman M.J., Stone H.A., Whitesides G.M. (2006). Formation of droplets and bubbles in a microfluidic T-junction-scaling and mechanism of break-up. Lab Chip.

[83] Pollack M.G., Shenderov A.D., Fair R.B. (2002). Electrowetting-based actuation of droplets for integrated microfluidics. Lab Chip.

[84] Berge B., Peseux J. (2000). Variable focal lens controlled by an external voltage: An application of electrowetting. Eur. Phys. J. E.

[85] Ahmadi A., Devlin K.D., Najjaran H., Holzman J.F., Hoorfar M. (2010). *In situ* characterization of microdroplet interfacial properties in digital microfluidic systems. Lab Chip.

[86] Ng J.M.K., Gitlin I., Stroock A.D., Whitesides G.M. (2002). Components for integrated poly(dimethylsiloxane) microfluidic systems. Electrophoresis.

[87] Becker H., Locascio L.E. (2002). Polymer microfluidic devices. Talanta.

[88] Sia S.K., Whitesides G.M. (2003). Microfluidic devices fabricated in poly(dimethylsiloxane) for biological studies. Electrophoresis.

[89] Feltis B.N., Sexton B.A., Glenn F.L., Best M.J., Wilkins M., Davis T.J. (2008). A hand-held surface plasmon resonance biosensor for the detection of ricin and other biological agents. Biosens. Bioelectron..

[90] Nie Z., Nijhuis C.A., Gong J., Chen X., Kumachev A., Martinez A.W., Narovlyansky M., Whitesides G.M. (2010). Electrochemical sensing in paper-based microfluidic devices. Lab Chip.

[91] Jing G., Polaczyk A., Oerther D.B., Papautsky I. (2007). Development of a microfluidic biosensor for detection of environmental mycobacteria. Sens. Actuators B Chem..

[92] Mark D., Haeberle S., Roth G., von Stetten F., Zengerle R. (2010). Microfluidic lab-on-a-chip platforms: Requirements, characteristics and applications. Chem. Soc. Rev..

[93] Whitesides G.M. (2006). The origins and the future of microfluidics. Nature.

[94] Bashir R. (2001). Microfuidic Biochip for Impedance Spectroscopy of Biological Species. Biomed. Microdevices.

[95] Weibel D.B., Whitesides G.M. (2006). Applications of microfluidics in chemical biology. Curr. Opin. Chem. Biol..

[96] Hong J., Edel J.B., deMello A.J. (2009). Micro- and nanofluidic systems for high-throughput biological screening. Drug Discov. Today.

[97] Bringer M.R., Gerdts C.J., Song H., Tice J.D., Ismagilov R.F. (2004). Microfluidic systems for chemical kinetics that rely on chaotic mixing in droplets. Philos. Trans. A. Math. Phys. Eng. Sci..

[98] Squires T.M. (2005). Microfluidics: Fluid physics at the nanoliter scale. Rev. Mod. Phys..

[99] Choi S., Chae J. (2009). A regenerative biosensing surface in microfluidics using electrochemical desorption of short-chain self-assembled monolayer. Microfluid. Nanofluidics.

[100] Auroux P.-A., Iossifidis D., Reyes D.R., Manz A. (2002). Micro Total Analysis Systems. 2. Analytical Standard Operations and Applications. Anal. Chem..

[101] Manz A., Graber N., Widmer H.M. (1990). Miniaturized total chemical analysis systems: A novel concept for chemical sensing. Sens. Actuators B Chem..

[102] Stone H.A., Kim S. (2001). Microfluidics: Basic issues, applications, and challenges. AIChE J..

[103] Anwar K., Han T., Yu S., Kim S. (2010). An Integrated Micro-Nanofluidic system for sample preparation and preconcentration of proteins. R. Soc. Chem..

[104] Liu K.-K., Wu R.-G., Chuang Y.-J., Khoo H.S., Huang S.-H., Tseng F.-G. (2010). Microfluidic systems for biosensing. Sensors.

[105] Wu J., Zheng G., Lee L.M. (2012). Optical imaging techniques in microfluidics and their applications. Lab Chip.

[106] Breslauer D.N., Lee P.J., Lee L.P. (2006). Microfluidics-based systems biology. Mol. Biosyst..

[107] Sato K., Mawatari K., Kitamori T. (2008). Microchip-based cell analysis and clinical diagnosis system. Lab Chip.

[108] Kim Y.-H., Sung H.J., Kim S., Kim E.-O., Lee J.W., Moon J.Y., Choi K., Jung J.-E., Lee Y., Koh S.S. (2011). An RNA aptamer that specifically binds pancreatic adenocarcinoma up-regulated factor inhibits migration and growth of pancreatic cancer cells. Cancer Lett..

[109] Gardeniers J.G.E., van den Berg A. (2004). Lab-on-a-chip systems for biomedical and environmental monitoring. Anal. Bioanal. Chem..

[110] Ali M.A., Srivastava S., Solanki P.R., Reddy V., Agrawal V.V., Kim C., John R., Malhotra B.D. (2013). Highly efficient bienzyme functionalized nanocomposite-based microfluidics biosensor platform for biomedical application. Sci. Rep..

[111] Lee K.-H., Su Y.-D., Chen S.-J., Tseng F.-G., Lee G.-B. (2007). Microfluidic systems integrated with two-dimensional surface plasmon resonance phase imaging systems for microarray immunoassay. Biosens. Bioelectron..

[112] Xu Y., Yang X., Wang E. (2010). Review: Aptamers in microfluidic chips. Anal. Chim. Acta.

[113] Zhou Q., Kwa T., Gao Y., Liu Y., Rahimian A., Revzin A. (2014). On-chip regeneration of aptasensors for monitoring cell secretion. Lab Chip.

[114] Ali-Cherif A., Begolo S., Descroix S., Viovy J.L., Malaquin L. (2012). Programmable magnetic tweezers and droplet microfluidic device for high-throughput nanoliter multi-step assays. Angew. Chemie - Int. Ed..

[115] Gu S., Lu Y., Ding Y., Li L., Song H., Wang J., Wu Q. (2014). A droplet-based microfluidic electrochemical sensor using platinum-black microelectrode and its application in high sensitive glucose sensing. Biosens. Bioelectron..

[116] Golberg A., Linshiz G., Kravets I., Stawski N., Hillson N.J., Yarmush M.L., Marks R.S., Konry T. (2014). Cloud-enabled microscopy and droplet microfluidic platform for specific detection of *Escherichia coli* in water. PLoS ONE.

[117] Iwai K., Sochol R.D., Lee L.P., Lin L. Finger-powered bead-in-droplet microfluidic system for point-of-care diagnostics. Proceedings of the 2012 IEEE 25th International Conference on Micro Electro Mechanical Systems (MEMS).

[118] Li L., Wang Q., Feng J., Tong L., Tang B. (2014). Highly sensitive and homogeneous detection of membrane protein on a single living cell by aptamer and nicking enzyme assisted signal amplification based on microfluidic droplets. Anal. Chem..

[119] Yu Y., Chen J., Zhou J. (2014). Parallel-plate lab-on-a-chip based on digital microfluidics for on-chip electrochemical analysis. J. Micromech. Microeng..

[120] Cho S.K., Fan S., Moon H., Angeles L. Towards digital microfluidic circuits creating, transporting, cutting and merging liquid droplets by electrowetting-based actuation. Proceedings of the Fifteenth IEEE International Conference on Micro Electro Mechanical Systems.

[121] Vergauwe N., Witters D., Ceyssens F., Vermeir S., Verbruggen B., Puers R., Lammertyn J. (2011). A versatile electrowetting-based digital microfluidic platform for quantitative homogeneous and heterogeneous bio-assays. J. Micromech. Microeng..

[122] Srinivasan V., Pamula V., Fair R. A Digital microfluidic biosensor for multianalyte detection. Proceedings of the 2003 IEEE The Sixteenth Annual International Conference on Micro Electro Mechanical Systems.

[123] Choi K., Kim J.-Y., Ahn J.-H., Choi J.-M., Im M., Choi Y.-K. (2012). Integration of field effect transistor-based biosensors with a digital microfluidic device for a lab-on-a-chip application. Lab Chip.

[124] García-Alonso J., Greenway G.M., Hardege J.D., Haswell S.J. (2009). A prototype microfluidic chip using fluorescent yeast for detection of toxic compounds. Biosens. Bioelectron..

